# Development of genetic tools for *Myceliophthora thermophila*

**DOI:** 10.1186/s12896-015-0165-5

**Published:** 2015-05-27

**Authors:** Jing Xu, Jingen Li, Liangcai Lin, Qian Liu, Wenliang Sun, Bangquan Huang, Chaoguang Tian

**Affiliations:** College of Life Sciences, Hubei University, Wuhan, 430062 China; Key Laboratory of Systems Microbial Biotechnology, Tianjin Institute of Industrial Biotechnology, Chinese Academy of Sciences, Tianjin, 300308 China

**Keywords:** *Myceliophthora thermophila*, *Agrobacterium tumefaciens*, genetic transformation, *ku70*

## Abstract

**Background:**

The thermophilic filamentous fungus *Myceliophthora thermophila* has many suitable characteristics for industrial biotechnology and could be a promising new chassis system for synthetic biology, particularly the ATCC 42464 strain, whose genome was sequenced in 2011. However, metabolic engineering of this strain using genetic approaches has not been reported owing to a lack of genetic tools for this organism.

**Results:**

In the present study, we developed a high efficiency *Agrobacterium tumefaciens* mediated transformation system for *M. thermophila*, including an approach for targeted gene deletion using green fluorescence protein (GFP) as a marker for selection. Up to 145 transformants per 10^5^ conidia were obtained in one transformation plate*.* Moreover, a *ku70* deletion mutant was constructed in the ATCC 42464 background using the tools developed in present study and subsequently characterized. The *ku70* deletion construct was designed using resistance to phosphinothricin as the selection marker. Additionally, a GFP-encoding cassette was incorporated that allowed for the selection of site-specific (no fluorescence) or ectopic (fluorescence) integration of the *ku70* construct. Transformants with ectopically integrated *ku70* deletion constructs were therefore identified using the fluorescent signal of GFP. PCR and Southern blotting analyses of non-fluorescent putative *ku70* deletion transformants revealed all 11 tested transformants to be correct deletions. The deletion frequency in a pool of 116 transformants analyzed was 58 %. Moreover, the homologous rate improved about 3 folds under *ku70* mutant using the *pyrG* as a test gene to disrupt in *M. thermophila*.

**Conclusions:**

We successfully developed an efficient transformation and target gene disruption approach for *M. thermophila* ATCC 42464 mediated by *A. tumefaciens.* The tools and the *ku70* deletion strain developed here should advance the development of *M. thermophila* as an industrial host through metabolic engineering and accelerate the elucidation of the mechanism of rapid cellulose degradation in this thermophilic fungus.

## Background

The thermophilic filamentous fungus *Myceliophthora thermophila* exhibits a large capacity for biomass degradation and represents a potential reservoir of novel enzymes for industrial applications, including abundant thermostable enzymes for biomass degradation [1–5]. *M. thermophila* also has the potential to be a cell factory to produce chemicals and biofuels from renewable lignocellulose [5]. Therefore, metabolic engineering of *M. thermophila* is attractive. To use *M. thermophila* in industrial biotechnology, the development of versatile genetic tools is urgently needed.

PEG-mediated protoplast transformation was developed to knock out target genes and for hetero-expression of interesting genes in several filamentous fungi, such as *Aspergillus* and *Trichoderma* [6–8]. It was previously reported this approach can be used to transform the industrial strain C1, previously known as *Chrysosporium lucknowense*, and recently renamed as an isolate of *Myceliophthora thermophila* [9]. However, preparation of fungal protoplasts was laborious, and heterokaryotic transformants might have genetic stability concerns [10, 11]. The C1 strain is a patented strain, therefore the ATCC 42464 strain is predominantly used for public research as the general wild-type strain of *M. thermophila*. The whole genome sequence of ATCC 42464 has been completed and is open to the public [12]. Various studies have been performed with *M. thermophila* ATCC 42464, such as biochemical characterization of enzymes [13–15] and investigating cellulose degradation on a cellular and genomic level [4, 12, 16, 17]. However, molecular engineering using a genetic approach has not been reported in *M. thermophila* ATCC 42464 because of a lack of mature genetic tools. *Agrobacterium tumefaciens*-mediated transformation has become a common technique for filamentous fungi [18–22]. This technique allows transfer of a segment from its Ti plasmid into plant or fungi cells, and the T-DNA integrates into the nuclear chromosome either randomly or specifically. The direct transformation of conidia makes *A. tumefaciens* T-DNA transfer a powerful and easy to use tool for genetic transformation of filamentous fungi. A variety of fungi have been successfully transformed by this approach [18–22]. However, the application of this approach in thermophilic filamentous fungi, including *M. thermophila*, has not been tested so far.

In present study, we developed a high efficiency transformation and targeted gene disruption system mediated by *A. tumefaciens* for *M. thermophila* ATCC 42464. Using this technique, up to 145 transformants were obtained per transformation plate, and a *ku70* deletion strain was constructed in the ATCC 42464 background and characterized. All of the tools and strains developed here should facilitate the development of *M. thermophila* metabolic engineering and accelerate the elucidation of the fascinating cellulose degradation mechanism in this thermophilic fungus.

## Results and discussion

### Marker selection and genetic transformation in the ATCC 42464 strain

To perform *M. thermophila* transformation mediated by *A. tumefaciens*, the sensitivity of this fungus to hygromycin B (Amresco, Solon, OH, USA) and phosphinothricin (Sigma-Aldrich, St. Louis, MO, USA) was tested. Five concentrations of two antibiotics were used. The mature conidia were harvested in 0.05 % Tween 80, and 10^3^ conidia were spread on MM (Vogel’s minimal medium supplemented with 2 % sucrose) agar plates supplemented with various concentrations of antibiotic (Hygromycin B: 25, 50, 75, 100, 150 μg/mL; phosphinothricin: 25, 50, 100, 150, 200 μg/mL). After incubation at 45 °C for 5 days, Hygromycin B showed little inhibition for *M. thermophila* at more than 100 ug/mL, whereas phosphinothricin concentration completely inhibited the growth of this fungus at 100 ug/mL. Thus, the *bar* gene conferring fungal resistance to phosphinothricin was employed as the selection marker for *M. thermophila* transformation.

**Fig. 1:**
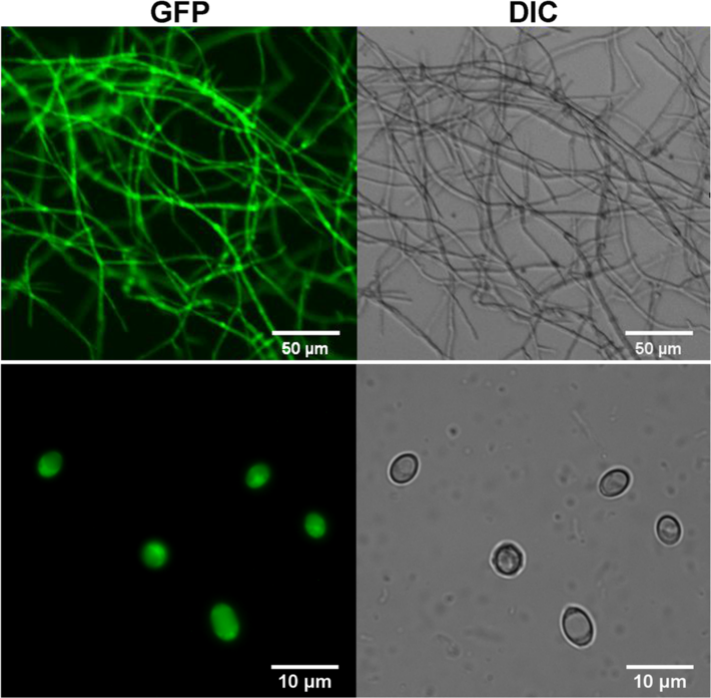
Confocal fluorescence imaging of mycelia (Top) and conidia (Bottom) of *M. thermophila* pPK2BarGFPD transformants

The Ti vector pPK2BarGFPD containing the *trpC* promoter from *Aspergillus nidulans* and the *tef* promoter from *Aureobasidium pullulans* (PtrpC and Ptef, respectively) developed in a previous study [23] was chosen as the test plasmid for genetic transformation in *M. thermophila* ATCC 42464. The vector contains a GFP reporter gene as well as a *bar* selection marker gene, which confers resistance to phosphinothricin. PCR analysis of the transformants showed the T-DNA was integrated in the chromosomes. High levels of green fluorescent protein (GFP) signal were clearly detected in the conidia and mycelia of transformants (Fig. 1), whereas no autofluorescent signal was observed in the parental strain. This suggested pPK2BarGFPD and its elements, the promoters and the GFP gene, were functioning and could be used for gene over-expression and protein localization analysis in *M. thermophila* ATCC 42464.

### Optimization of *M. thermophila* transformation mediated by *A. tumefaciens*

**Fig. 2:**
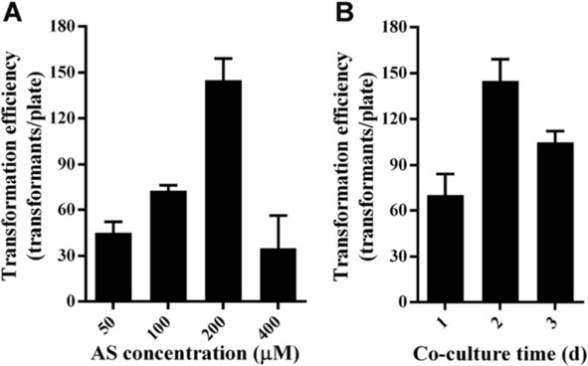
Optimization of *A. tumefaciens*-mediated *M. thermophila* transformation. **a** The impact of acetosyringone (AS) on *A. tumefaciens*-mediated transformation efficiency. Co-cultivation was conducted in induction medium containing AS on indicated concentrations for 2 days. **b** The effect of co-culture time on transformation efficiency. Co-cultivation was conducted in induction medium containing 200 μM AS for various times indicated. Error bars indicate the standard deviation of three independent experiments

In an attempt to develop a simple, high efficiency transformation system mediated by *A. tumefaciens* in *M. thermophila* ATCC 42464, we performed transformation optimization as described previously [21]. Acetosyringone (AS) serves as an inducer of *virulence (vir)* genes, whose expression is a prerequisite for *A. tumefaciens* T-DNA transfer. AS concentration influences the frequency of *A. tumefaciens*-mediated transformation for *Saccharomyces cerevisiae* and several filamentous fungi [11, 20, 24, 25]. Therefore, the impact of AS on this approach for *M. thermophila* was first investigated. As shown in Fig. 2a, using the binary plasmid pPK2BarGFPD, the maximum transformation efficiency (145 ± 10 transformants per plate) was obtained with AS at a concentration of 200 μM, and the number of transformants was reduced when the concentration was increased or decreased. The observation that the higher concentration of AS resulted in a reduction in transformation efficiency is similar to that for *Fusarium avenaceum*. However, it is in contrast to that for *Beauveria bassiana*, where an increase in the concentration from 100 μM to 800 μM resulted in increasing numbers of transformants [21, 26]. Based on these observation, the different fungus seems has different tolerance to Acetosyringone (AS), the low frequency of transformation induced by the increased AS might be attributed to the toxic effect of AS on *M. thermophila*, and the deficient level of *vir* gene expression at low AS concentrations might be responsible for poor efficiency of *A. tumefaciens* T-DNA transfer.

The transformation efficiency is also dependent on co-culture time of *M. thermophila* and *A. tumefaciens*. The largest number of transformants was observed with a co-culture time of 2 days, and alterative co-culture times impaired the transformation efficiency (Fig. 2b). Similar results were obtained in *Metarhizium anisopliae, Aspergillus terreus* and *Beauveria bassiana* transformation mediated by *A. tumefaciens* [2, 18, 27], whereas the co-culture time had no influence on a *Ganoderma lucidum* transformation system [28]. In conclusion, with the optimized approach, a high transformation efficiency of 145 transformants per 10^5^ conidia was obtained. This was higher compared with the amount of transformants obtained in fungi using other approaches, typically less than 100 transformants per 10^5^ conidia [18, 29–31].

### Mitotic stability analysis of transformants

The mitotic stability of the T-DNA in *M. thermophila* transformants was examined next. Twenty randomly selected transformants containing pPK2BarGFPD were cultured on media without phosphinothricin for five generations. After five generations, 19 transformants (95 %) of 20 tested strains retained the resistance to phosphinothricin. Satisfyingly, PCR analysis showed the presence of *bar* in the 19 transformants, and GFP signal was observed under the fluorescence microscope.

### *ku70* disruption of *M. thermophila* ATCC 42464

The high transformation frequency, together with the precision and simplicity of T-DNA, make *A. tumefaciens*-mediated transformation a suitable genome mutagenesis approach in filamentous fungi, such as *Trichoderma*, *Aspergillus*, *Beauveria* and *Metarhizium* [22, 27, 32, 33]. Ku70 and Ku80 make up the Ku heterodimer, which binds to the ends of double-stranded DNA breaks and is required for the non-homologous end joining (NHEJ) pathway of DNA repair [34]. In some fungi, including the *M. thermophila* C1 strain, *Trichoderma reesei* and *Neurospora crassa*, mutation of *ku70* results in a dramatically increased homologous integration frequency with short homologous flanks [9, 35, 36]. Therefore, disruption of *ku70* of the ATCC 42464 strain was performed to test the efficiency of this approach for knocking out of a specific gene in wild-type *M. thermophila*.

**Fig. 3:**
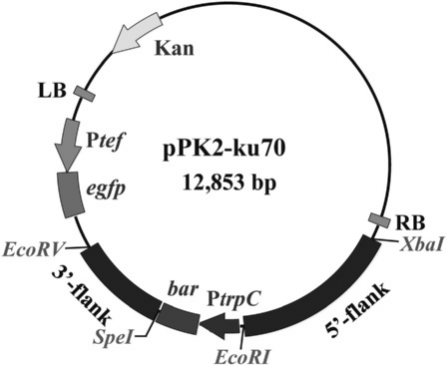
Vector map of the pPK2-ku70 vector constructed based on binary vector pPK2BarGFPD. Kan, kanamycin resistance gene; P*tef*, promoter of translation elongation factor gene from *Aureobasidium pullulans*; *egfp*, enhanced green fluorescence protein; P*trpC*, promoter of tryptophan synthetase gene from *Aspergillus nidulans*; *bar*, phosphinothricin resistance gene; 3′-flank and 5′-flank, 3′ and 5′ flanking fragments of *ku70*, respectively; RB and LB, right and left border of T-DNA, respectively

**Fig. 4:**
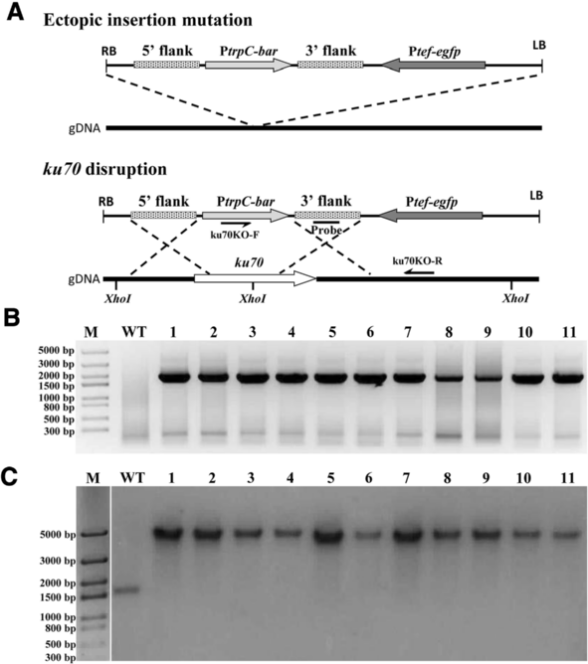
*ku70* disruption of wild-type *M. thermophila* ATCC 42464. **a** Pattern of the *ku70* knockout cassette integrating into the chromosomes of *M. thermophila* ATCC 42464 via ectopic insertion or homologous recombination. **b** PCR analysis of *ku70* deletion transformants with one primer (ku70KO-F) located in the *bar* gene cassette and the other (ku70KO-R) located in the downstream of 3′ flank in the genomic DNA (expected product of 1872 bp) and **c** Southern blotting analysis of *ku70* mutants with *Xho*I digested genomic DNA and the probe amplified from the 3′ flank. Lines 1–11 in (**b**) and (**c**), genomic DNA from *ku70* mutants (expected product of 5056 bp). WT, genomic DNA from wild-type as negative control (expected product of 1703 bp). Primers are represented by solid black arrows. Abbreviations: gDNA, genomic DNA; RB and LB, right and left border of T-DNA, respectively

The binary vector pPK2BarGFPD was employed for DNA manipulation and the *ku70* knockout cassette (Fig. 3) was constructed as described in the Methods section. The gene encoding green fluorescence protein on the binary vector served as the marker to differentiate between ectopic insertion and correct gene replacement. Fluorescence should not be detected in the transformants where the target gene (*ku70*) was properly replaced by the *bar* cassette (Fig. 4a). Conversely, if the fluorescent signal is detected in a transformant, this suggests the *bar-gfp* segment of the T-DNA was ectopically integrated elsewhere in the genome (random insertion) rather than at the correct gene locus. Using this strategy, we performed the *ku70* disruption experiment. One hundred sixteen primary transformants were obtained. Forty-six (39.7 %) transformants were filtered out by an obvious fluorescent signal, indicating they were ectopic insertion mutants. The remaining 70 transformants (60.3 %) with no detectable fluorescent signal under the microscope were likely free of ectopic insertions and passed the first round screening. Subsequent PCR analysis showed the *ku70* gene was properly replaced by the *bar* cassette in 68 of these 70 transformants. To further confirm this observation, we randomly selected eleven PCR-confirmed transformants to perform Southern blotting analysis, and the result showed all 11 tested mutants were correct (Fig. 4b and c). Taken together, these results indicate as many as 58 % (68 of 116) of the transformants could be correct *ku70* disruption mutants, indicating a very high rate of homologous recombination. Previous studies reported that the efficiencies of genetic targeted disruption via transformation mediated by *A. tumefaciens* varied greatly in fungal species, such as 0.04 % for *Blastomyces dermatitidis*, 29 % for *Aspergillus awamori*, 74 % for *Fusarium avenaceum* and 85 % for *Fusarium graminearum* [37, 38]. Compared with transformation by direct electroporation transformation of protoplasts or conidia, the transformation mediated by *A. tumefaciens* seems more likely to achieve a higher successful gene disruption rate, at least for some of the species. For example, in *Neurospora crassa* the homologous recombination rate was usually low (<10 %) without NHEJ disruption using electroporation [35]. Although future studies with more precise statistical analyses are needed to support the improved homologous recombination rate, if validated, it will be a boost for genetic manipulation in this strain, and will speed up strain engineering for industrial applications.

**Fig. 5:**
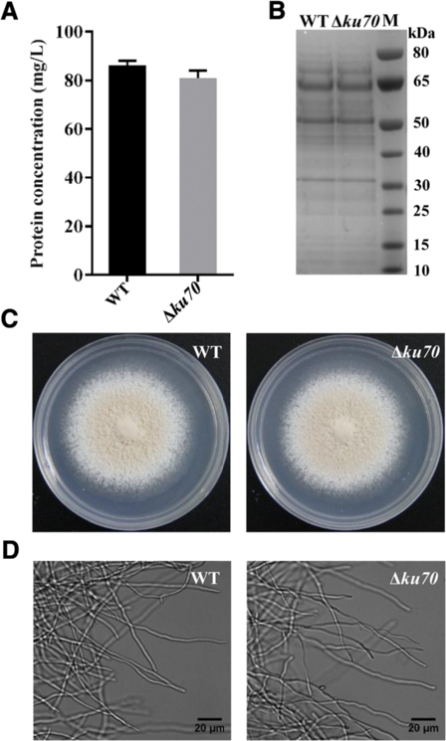
Phenotypic characterization of *ku70* mutant. **a** Protein concentration in supernatants from wild-type (WT) and ku70 mutant cultures grown on 1 % Avicel for 3 d at 45 °C. **b** SDS-PAGE gel of wild-type and *Δku70* strains grown at 45 °C for 3 d on 1 % Avicel. **c** Conidiation of wild-type and *Δku70* strains growth at 45 °C for 9 d on agar plate without selective agent. **d** The hyphae of wild-type and *Δku70* strains growth at 45 °C for 24 h on liquid MM without selective agent

There was no noticeable difference between *ku70* disruption mutants and the wild-type strain during our analysis (Fig. 5). After five generations of sub-culturing on MM agar medium, 10 randomly selected *ku70* mutants were shown to be mitotically stable. Taken together, these results indicate the approach we developed is an efficient system for gene disruption in *M. thermophila* ATCC 42464.

**Fig. 6:**
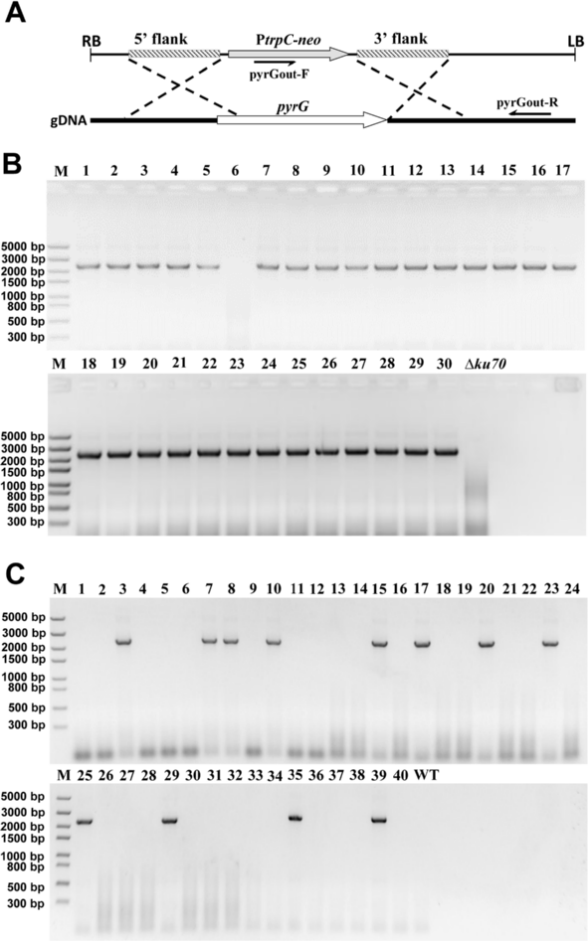
pyrG gene deletion in wild type and ∆ku70 mutant of *M. thermophila* ATCC 42464. The experiment design (**a**), PCR analysis of transformants under wild-type background (**b**) and ∆ku70 mutant (**c**) with one primer (pyrGout-F) located in the neo gene cassette and the other (pyrGout-R) located in the downstream of 3′ flank in the genomic DNA. The genomic DNA from the wild-type (WT) and ∆ku70 strains was used as the negative control

In order to check whether the gene homologous recombination rate was improved in *ku70* mutant compared to that in wild type strain, we chose the potential marker gene *pyrG* (MYCTH_2311494) as a test to disrupt in both wild-type and *ku70* deletion strain. As expected, 97 % (29/30) of transformants were correct *pyrG* deletion mutants under *ku70* background, whereas only 30 % (12/40) of transformants were correct in wild type background (Fig. 6). This result clearly shown the deletion of KU70 gene can improve the gene homologous recombination in this strain, similar as reported before [9].

Taken together, these results indicate the approach we developed is an efficient system for gene disruption in *M. thermophila* ATCC 42464 and will speed up its strain engineering for industrial applications.

## Conclusions

We successfully developed a simple and highly efficient transformation approach mediated by *A. tumefaciens* for the thermophilic filamentous fungus *M. thermophila*. This study established a targeted gene deletion approach using green fluorescence protein as a selection helper to filter the ectopic insertion. The tools and *ku70* mutant developed by the present study should facilitate *M. thermophila* strain development for industrial applications in the future.

## Methods

### Strains and culture conditions

*Myceliophthora thermophila* ATCC 42464 obtained from the American Type Culture Collection (ATCC) was grown on Vogel’s minimal medium supplemented with 2 % sucrose (MM medium) at 45 °C for 15 d to obtain conidia. Sucrose was purchased from Amresco (Solon, OH, USA).

*Escherichia coli* DH5α was used for plasmid amplification and vector construction and cultivated in Luria–Bertani medium with antibiotics according to conditions for plasmid selection.

*Agrobacterium tumefaciens* AGL-1 [23] used for *M. thermophila* transformation, was grown in Luria–Bertani medium at 28 °C and kanamycin was added when needed to maintain binary plasmids [39].

### Preparation and transformation of *M. thermophila* ATCC 42464 mediated by *Agrobacterium*

*M. thermophila* transformation mediated by *A. tumefaciens* was performed using a modification of the method described previously for *Fusarium circinatum* [21]. A single colony of *A. tumefaciens* harboring binary vector was inoculated into LB medium supplemented with 50 μg/mL kanamycin and cultivated with shaking of 250 rpm at 28 °C for 12–16 h to an optical density of 0.5–1.0 at 600 nm. Subsequently, cells were harvested, washed twice with induction medium (IM) containing AS and then diluted to an OD600 of 0.15. The culture was grown at 28 °C with shaking at 250 rpm for 6–8 h to an OD600 of 0.5–0.8. Conidia were harvested into 0.05 % Tween 80, filtered through glass wool to remove residual mycelia and adjusted to 10^6^ conidia per milliliter calculated using a hemocytometer. An aliquot of 100 μL *A. tumefaciens* culture was mixed with an equal volume of *M. thermophila* conidia and spread on an IM agar plate (9.0 cm) containing AS and was covered with cellophane. After co-cultivation at 28 °C, the cellophane was transferred onto MM medium plate supplemented with 100 μg/mL phosphinothricin (Sigma-Aldrich, St. Louis, USA) and 300 μg/mL cefotaxime (Sigma-Aldrich), overlaid with Vogel’s salt agar medium containing 2 % sucrose and 100 μg/mL phosphinothricin and then cultivated at 45 °C. Putative transformants were visible after approximately 3 days.

### Mitotic stability analysis

Enhanced GFP (EGFP) was used as the reporter gene to elucidate mitotic stability of transformants. Twenty randomly selected successful transformants with pPK2BarGFPD were consecutively sub-cultured on agar plates without the selective agent. When the agar plates were full of mycelia and conidia, conidia were used to inoculate the next generation culture. After five generations, the GFP-signal was detected under fluorescence microscopy and PCR analysis targeting the *bar* gene with the specific primer sets, bar-F/bar-R (Table 1), was performed. Triplicate reactions were performed for each transformant.

**Table 1:** Primers used for the manipulation of ku70 and the identification of its mutants

Names	Primer sequences (5′-3′)	Notes
bar-F	CTGGAGCTAGTGGAGGTCAAC	PCR detection of *bar*
bar-R	TTCAATCTTAAGAAACTTTATTG	PCR detection of *bar*
ku70-5 F	GCTCTAGAGCGCTACCACGGACGGATGATAC	Cloning *ku70* 5’flank
ku70-5R	CCGGAATTCGGCTTTGAAGGTGCAGGTGCGAC	Cloning *ku70* 5’flank
ku70-3 F	GGACTAGTGTTGGGATCGCATGGTTCGTTG	Cloning *ku70* 3’flank
ku70-3R	CCGATATCGGCTTCAGAATGCAGAGGTCAGAG	Cloning *ku70* 3’flank
Probe-F	GCGACGATCCCGAAATAC	Southern blotting of *ku70*
Probe-R	CCCTGACGAAGTAGCATCAT	Southern blotting of *ku70*
ku70KO-F	CTACACCCACCTGCTGAAGTCC	PCR detection of *ku70* mutation
ku70KO-R	GTCCCGCACTACCGTTGATGG	PCR detection of *ku70* mutation
ku70-F	CGTGGGAAGGTGACGATGATAGAC	PCR detection of *ku70* ORF
ku70-R	CATAATGCTCCTCGCTCGGGAAG	PCR detection of *ku70* ORF
PtrpC-F	AAAAAGCTTGGTACCGAGCTCCGACGTTAACTGATATTGAAGGAGC	Cloning *PtrpC*
PtrpC-R	CGTGCAATCCATCTTGTTCAATCATTTGGATGCTTGGGTAGAATAGGTAA	Cloning *PtrpC*
neo-F	TTACCTATTCTACCCAAGCATCCAAATGATTGAACAAGATGGATTGCACG	Cloning *neo*
neo-R	AAATTAATTAAGTTTAAACCTCGAGTCTAGAAGATCTTCAGAAGAACTCGTCAAGAAGGCGA	Cloning *neo*
pyrG-5 F	AAAGAATTCCGATGCAGATGCAACTCCGCTCCCT	Cloning *pyrG* 5’flank
pyrG-5R	AAAGGATCCAGGTTCGAGGCCTTGAGGTCCATGA	Cloning *pyrG* 5’flank
pyrG-3 F	CCGCTCGAGATCTGGCGCGTCTGGCGTGATTTGG	Cloning *pyrG* 3’flank
pyrG-3R	GGAAGATCTCGGGGTGAGTGTTGGGGTGTTGTTT	Cloning *pyrG* 3’flank
pyrGout-F	GGGCTGACCGCTTCCTCGTGCTTTA	PCR detection of *pyrG*
pyrGout-R	TGGCGTAGTGCGTGTTGAACTCGGC	PCR detection of *pyrG*

Underlined regions indicate restriction enzyme sites

### Binary vector construction for *ku70* disruption

The master Ti vector pPK2BarGFPD [23] containing the phosphinothricin resistant *bar* marker, was employed to test transformation efficiency of *M. thermophila* mediated by *A. tumefaciens*. To disrupt the *ku70* gene involved in the nonhomologous end-joining (NHEJ) pathway, the plasmid pPK2-ku70 was constructed (Fig. 3). The 5′ and 3′ flanking fragments (2095 bp and 1246 bp) were amplified from *M. thermophila* genomic DNA with paired primers (Table 1) designed using the annotated genome of *M. thermophila* ATCC 42464 [12]. The two flanks were inserted between the *Xba*I/*Eco*RI and *Spe*I/*Eco*RV sites of pPK2BarGFPD, respectively, to result in pPK2-ku70. The inserted fragments were sequenced to confirm the authenticity of the modified plasmids. All restriction enzymes were obtained from Thermo Scientific (Waltham, MA, USA).

### Identification of transformants by PCR and Southern blotting

Transformants harboring pPK2BarGFPD were verified by PCR analysis with paired primers, bar-F/bar-R (Table 1), to detect *bar* after fluorescence detection under the microscope.

The *ku70* deletion mutant transformants with ectopic plasmid insertions were recognized by checking fluorescence under the microscope. The green fluorescent signal suggested it was an ectopic insertion mutant rather than homologous recombination replacement. Proper gene replacement was confirmed by amplifying specific fragments with one primer located in the *bar* gene and the other located in the downstream of 3′ flank in the genomic DNA (Table 1). Mutants with correct placement of the *bar* gene underwent further homokaryon analysis. The knockout mutants containing no wild-type open reading fragment of *ku70* were determined by PCR analysis using paired primers, ku70-F/ku70-R (Table 1).

To verify the authenticity of *ku70* mutants, Southern blotting was performed with 20 μg genomic DNA digested by *Xho*I. Genomic DNA was extracted as described previously [40]. The digested DNA was separated by agarose gel electrophoresis and DNA transfer was performed as described previously [41]. A 441 bp PCR-amplified product used as the probe was generated with paired primers, Probe-F/Probe-R (Table 1). Probe preparation, membrane hybridization and visualization were performed according to the manufacturer’s instructions (DIG High Prime DNA Labeling and Detection Starter Kit II, Roche, Mannheim, Germany).

### *pyrG* disription in *ku70* mutant

The pyrG gene (MYCTH_2311494) encoding orotidine-5'-phosphate decarboxylase was disrupted through homologous replacement in the wild-type strain (WT) and *ku70* mutant. The P*trpC* and neomycin resistant elements were amplified from pCB1003 and pEGFP-N2 using paired primers PtrpC-F/R and neo-F/R, respectively, and fused using the primers PtrpC-F/neo-R with several restriction sites introduced to both ends. The PtrpC-neo cassette was cloned into the *Hind*III-*Bgl*II sites of pCAMBIA-0380 (CAMBIA, Canberra, Australia; p0380 herein) to generate p0380-neo. The 5′ and 3′ fragments of *pyrG* (2071 and 1972 bp) were separately amplified from *M. thernophila* genomic DNA via PCR with paired primers (Table 1). The amplified fragments were inserted into *Eco*RI/*Bam*HI and *Xho*I/*Bgl*II sites in p0380-neo plasmid, forming the disruption plasmid p0380-pyrGup-neo-pyrGdn. The constructed plasmids were transformed into WT and *ku70* deletion strains via *Agrobacterium*-mediated transformation. Colonies grown for 5 or 6 days on a selective medium at 45 °C were screened in terms of the neomycin resistance to G418 (40 μg mL/L), followed by identification via PCR with paired primers (Table 1).

### Microscopy

Confocal microscopy was performed on a Leica TCS SP5 II microscope using a 100× 1.4 NA oil or 20× immersion objective, 30 % Laser (Argon, visible) Power, PMT Trans of 180 HV, Emission bandwidth of PMT was 500 nm to 600 nm. The images were processed and analyzed by ImageJ (version 1.47).

### Phenotype characterization of the *ku70* mutant

Mature conidia from wild-type and *ku70* mutant strains were inoculated into 100 mL 1 × Vogel’s salt supplemented with 1 % Avicel (PH101, Sigma-Aldrich, St. Louis, MO, USA) at 2.5 × 10^5^ conidia per milliliter in a 250-mL Erlenmeyer flask. After growth for 16 h at 45 °C with shaking at 150 rpm under constant light, total secreted protein in the supernatant was determined using a Bio-Rad DC protein assay kit (Bio-Rad, Hercules, CA, USA) and 25 μl of supernatant was used for SDS-PAGE analysis. The experiments were performed in biological triplicates.

## Abbreviations

AS: Acetosyringone
EGFP: Enhanced green fluorescent protein
DIC: Differential interference contrast
gDNA: Genomic DNA
IM: Induction medium
NHEJ: Nonhomologous end-joining
PCR: Polymerase chain reaction
RB and LB: Right and left border of T-DNA, respectively
